# Identification of Personalized Chemoresistance Genes in Subtypes of Basal-Like Breast Cancer Based on Functional Differences Using Pathway Analysis

**DOI:** 10.1371/journal.pone.0131183

**Published:** 2015-06-30

**Authors:** Tong Wu, Xudong Wang, Jing Li, Xiuzhen Song, Ying Wang, Yunfeng Wang, Lei Zhang, Ziyao Li, Jiawei Tian

**Affiliations:** 1 Department of Ultrasound, the Second Affiliated Hospital of Harbin Medical University, Harbin City, Heilongjiang Province, China; 2 Department of Ultrasound, the First Hospital of Qiqihar, Qiqihar City, Heilongjiang Province, China; 3 Department of Ultrasound, the Frontier Corps Hospital in Heilongjiang Province, Harbin City, Heilongjiang Province, China; 4 Department of General Surgery, the Second Hospital of Hebei Medical University, Shijiazhuang City, Hebei Province, China; 5 College of Bioinformatics Science and Technology, Harbin Medical University, Harbin City, Heilongjiang Province, China; University of North Carolina School of Medicine, UNITED STATES

## Abstract

Breast cancer is a highly heterogeneous disease that is clinically classified into several subtypes. Among these subtypes, basal-like breast cancer largely overlaps with triple-negative breast cancer (TNBC), and these two groups are generally studied together as a single entity. Differences in the molecular makeup of breast cancers can result in different treatment strategies and prognoses for patients with different breast cancer subtypes. Compared with other subtypes, basal-like and other ER+ breast cancer subtypes exhibit marked differences in etiologic factors, clinical characteristics and therapeutic potential. Anthracycline drugs are typically used as the first-line clinical treatment for basal-like breast cancer subtypes. However, certain patients develop drug resistance following chemotherapy, which can lead to disease relapse and death. Even among patients with basal-like breast cancer, there can be significant molecular differences, and it is difficult to identify specific drug resistance proteins in any given patient using conventional variance testing methods. Therefore, we designed a new method for identifying drug resistance genes. Subgroups, personalized biomarkers, and therapy targets were identified using cluster analysis of differentially expressed genes. We found that basal-like breast cancer could be further divided into at least four distinct subgroups, including two groups at risk for drug resistance and two groups characterized by sensitivity to pharmacotherapy. Based on functional differences among these subgroups, we identified nine biomarkers related to drug resistance: SYK, LCK, GAB2, PAWR, PPARG, MDFI, ZAP70, CIITA and ACTA1. Finally, based on the deviation scores of the examined pathways, 16 pathways were shown to exhibit varying degrees of abnormality in the various subgroups, indicating that patients with different subtypes of basal-like breast cancer can be characterized by differences in the functional status of these pathways. Therefore, these nine differentially expressed genes and their associated functional pathways should provide the basis for novel personalized clinical treatments of basal-like breast cancer.

## Introduction

Breast cancer is highly heterogeneous and most frequently occurs in females. This disease is divided into several different clinical subtypes, including luminal A, luminal B, basal- and normal-like, based on differences in gene expression profiling and immunohistochemical indicators. Among these subtypes, basal-like breast cancer largely overlaps with triple-negative breast cancer (TNBC). Therefore, basal-like breast cancer is usually described as a form of TNBC, and these diseases are commonly studied together as a single group [1]. TNBC is an aggressive subtype of breast cancer that is defined by the absence of ER, PR and HER2, and it is characterized by poor prognosis and rarely benefits from targeted therapy [2,3]. Effective targeted therapies specific for TNBC currently do not exist, and treatment regimens for TNBC are limited [4]. Hence, elucidating the mechanisms underlying resistance to adjunctive chemotherapy drugs and identifying new biomarkers and potential therapeutic targets in TNBC patients remain significant and challenging goals for modern clinical practice.

Drug resistance is commonly observed in TNBC patients and is more common than in non-TNBC patients [5,6]. Studies have revealed numerous drug resistance mechanisms in TNBC patients, and multiple genes and biological pathways have been implicated in this process. For example, CD73 and CD133 have been shown to impact drug-mediated anti-tumor immune responses, and IMP3 regulates the drug resistance proteins, ABCG2 and HSF1, as well as autophagy related protein 7 (ATG7) [4,7–9]. The PI3K/AKT/mTOR pathways have also been linked to drug resistance [10] through the regulation of multiple biological processes in the human body.

To realize the possibility of personalized therapy in TNBC patients, much research has been devoted to identifying personalized signatures by subdividing TNBC patients into subgroups that present different molecular characteristics or prognoses. One of the most significant works in this field was that of Lehmann and colleagues [3]. Lehmann et al. identified six TNBC subgroups using K-means clustering by amassing TNBC patient data from multiple platforms. They demonstrated that TNBC can be divided into distinct subgroups, each of which has distinct molecular characteristics. However, whether chemotherapy response was significantly different between these six subgroups was not addressed in detail. To efficiently identify prognosis signatures, Ke-Da Yul [11] treated the chemotherapy sensitive and resistant groups as two independent subgroups of TNBC, eventually identifying seven gene prognosis signatures. The purpose of this study was to integrate the main ideas of Lehmann and Ke-Da Yu to take into account both heterogeneity among TNBC subgroups and personalized resistant biomarkers in TNBC patients.

One commonly used method to identify important specific genes that mediate a given phenotype is to determine the gene expression signature of the case group and then compare this with a control group. Genes that exhibit statistically significant differences in expression between these two groups are then potentially linked to the phenotype of the case group. Conventional variance test methods, such as t tests, Mann-Whitney tests, and the significance analysis of Microarray (SAM) approach [12], have numerous advantages for analyzing homogeneous tumors. However, these methods are not suitable for highly heterogeneous tumors such as breast cancer. In particular, conventional variance test methods lack the sensitivity to identify personalized drug resistance genes when comparing breast cancer patients as a single group to controls. Indeed, potential specific biomarker genes are likely to only exhibit significant differential expression within particular subgroups, where they could play important roles in the individualized development of drug resistance [13,14].

To identify these personalized genes, we developed an algorithm to score the differential expression of each gene between the sensitive group and the drug-resistant group and then verify significant genes via random perturbation. In addition to differentially expressed genes that would have been identified using conventional variance tests, this new method can also effectively identify personalized genes that are differentially expressed in specific subgroups of the case group. Next, the identified genes were subjected to cluster analysis and enrichment analysis. Finally, based on molecular correlations, basal-like cancer patients were divided into two high risky subgroups and two low risky subgroups, and drug-resistance-related genes and pathways were identified for each subgroup. This analysis allowed us to quantify the degree of similarity and specificity for the mechanisms of drug resistance between the different subgroups. Each subgroup showed a specific set of differentially expressed genes and pathways, although several common drug resistance genes and biological pathways were shared between multiple subgroups. It is possible that these subgroups share the same terminal target, and certain drug-related proteins may eventually be affected in all subgroups, despite the fact that different upstream biological processes are involved. The nine identified genes represent potential biomarkers and targets for personalized clinical treatment, and they may help to improve clinical efficacy and reduce the side effects of anti-cancer drugs.

## Materials and Methods

### Discovery cohort

A total of 178 samples were downloaded from the expression profile dataset GSE34138 in the GEO database, including 46 basal-like subtype patients (24 drug-sensitive patients and 22 drug-resistant patients), 68 luminal A subtype patients (four drug-sensitive patients and 64 drug-resistant patients), 44 luminal B subtype patients (six drug-sensitive patients and 38 drug-resistant patients), and 20 normal-like and Her2 type patients. As we aimed to study the personalized resistant biomarkers for the Basal (HER2 negative) type of breast cancer, we removed the 20 normal-like and Her2 type patients.

### Validation cohort

To demonstrate that the resistant-related genes identified here have portability and repeatability, we obtained a validation cohort from the GEO and EBI databases (GSE1456, GSE3494, E-TABM-158). A total of 93 TNBC patients were screened based on ER, PR or HER2 status. The validation data were normalized using RMA methods and then integrated into one profile. Considering that the chemoresistance-induced relapse ratio was highest during the first 3 years [11], we treated patients with DSS Time (Disease-Specific Survival Time in years) values less than 3 years as chemoresistant and those with DSS Time values greater than 3 years as chemosensitive.

### Extraction of differentially expressed genes

Data from breast cancer samples of the luminal and basal-like subtypes were first selected from the expression profile dataset GSE34138. Due to intrinsic molecular variations between the samples of these two subtypes, the 46 basal-like subtype samples and 112 luminal subtype samples were analyzed separately to maintain sample homogeneity. The two sets of expression profiles were normalized using the RMA method to eliminate inherent differences in the levels of gene expression. All expression values were subject to Z-score corrections. In the normalized expression profiles, samples that were sensitive to chemotherapy (CR) were assigned to the sensitive group and those that were insensitive to chemotherapy (NOCR) were assigned to the drug-resistant group. The normal range was defined based on the expression values of the sensitive group (mean ± standard deviation). Effectively, this meant that the expression values of the patients in the sensitive group were defined as fluctuating within the normal range and that drug resistance was likely to occur when a patient expression values were outside that normal range. The number of samples in the sensitive group was defined as n1 and the number of samples in the drug-resistant group was defined as n2, and the score of gene g was calculated for expression outside the normal range for all drug-resistant groups using Formula 1.

Formula 1. Calculation of deviation score

$$\begin{array}{l} \;\text{score}=\;\sum_{i=1}^{n2}(X_{2i}-X\prime )\\ \text{where}\\ \;X\prime \;=\{\begin{array}{c} \;X_{max}\;if\;X_{2i}>X_{max}\\ X_{2i}\;if\;X_{min}<X_{2i}<X_{max}\\ \;X_{min}\;if\;X_{2i}<X_{min} \end{array} \end{array}$$ (1)

In this formula, X_2i_ represents the expression value of gene g in patient I in the drug-resistant group, and X_max_ and X_min_ represents the two limit values of the normal range. The raw score of gene g was calculated using the cumulative sum of the scores for gene g in the n2 patients in the drug-resistant group.

After obtaining the raw score for gene g, the n1 samples in the sensitive group and the n2 samples in the drug-resistant group were subject to 10,000 random permutations, and the n1 samples were then randomly selected as the sensitive group, with the remaining n2 samples counting as the drug-resistant group. Then, a new random score was calculated using Formula 1. The above procedure was repeated 10,000 times to acquire the background distribution of scores for gene g, which was then converted to a P value. A gene was considered to be significantly different between the groups when P<0.05.

### Hierarchical cluster analysis

Cluster analysis was performed to examine the extracted genes with significantly different levels of expression between patients with diverse outcomes in the luminal and basal-like subtypes. Average linkage hierarchical cluster analysis using Pearson correlations was conducted using the Cluster 3.0 program, and the data were visualized in Treeview [15,16]. The expression profiling data were filtered and standardized using the Cluster 3.0 program, and the genes and samples were standardized using the median center method. Centered correlation was used in the similarity matrix, and hierarchical clustering was used for the cluster analysis. The analyses were visualized as heat maps. As luminal type breast cancer can be divided into two subgroups, luminal A and luminal B [17], the differentially expressed genes in luminal breast cancer patients were extracted for cluster analysis to evaluate the efficacy of the proposed method for subdividing this single breast cancer subtype into personalized subgroups. The basal-like type of breast cancer was then analyzed to identify intrinsic subgroups.

### Allocation of subgroup-specific genes

Samples that had similar molecular expression profiles were clustered together using hierarchical clustering. In the drug-resistant group, patients in different subgroups may share similar specific drug resistance mechanisms. By comparing the specific expression patterns in each subgroup, candidate genes were allocated into each subgroup. Assuming a total of m subgroups were obtained in the drug-resistant group and n subgroups in the sensitive group via hierarchical clustering, determination of whether a gene was differentially expressed in a specific subgroup was made by calculating the mean value of this gene in the subgroups of the drug-resistant group (x1, x2…xm) and the mean value of this gene in the subgroups of the sensitive group (y1, y2…yn). The fluctuation range was then calculated based on the mean expression values of the drug-resistant group and the sensitive group. If the mean value for a given gene within the subgroups of the drug-resistant group was outside of the fluctuation range of the sensitive group, this indicated that the gene was differentially expressed in the subgroups of the drug-resistant group compared with the sensitive group; therefore, this gene was considered to be specific for the drug-resistant subgroup. However, when the mean value of a given gene within the subgroups of the sensitive group was outside the fluctuation range of the drug-resistant group, this indicated that the gene was stably expressed in the sensitive group and that its abnormal expression could lead to drug resistance; therefore, genes of this type were allocated to the sensitive subgroup.

### Identification of specific pathways and genes related to drug resistance

The corresponding specific gene set was obtained by allocating differentially expressed genes to various subgroups based on their mean expression values. These subgroup-specific genes exhibited significant differences in expression when compared with the sensitive group. Thus, they represent candidate genes that may be involved in drug resistance mechanisms in the different subgroups, and research into the functions of these specific genes and the biological processes they affect could be extremely useful for personalized clinical treatment. To analyze the biological processes in which a specific set of genes are involved, functional enrichment analysis was performed for the specific genes in each subgroup. KEGG pathway enrichment analysis was completed using the molecule annotation system V3.0 [18], and pathways with P values lower than 0.05 were considered to be statistically significant.

### Pathway deviation score

Functional annotation analysis was conducted on the specific gene set of the subgroups in the drug-resistant group. As specific genes exhibited different expression patterns in the different subgroups, the corresponding functional levels also varied. Functional pathways exhibiting differential expression levels in drug-resistant patients compared with sensitive patients could provide important clues for the development of personalized therapies for breast cancer. Hence, quantitative scoring of potential pathways was performed based on genes that were enriched in each potential pathway.

Formula 2.

$$\text{A}\left(\text{P}\right)\;=\text{lg}\left(\frac{1}{N}\sqrt{\sum_{i=1}^{N}{\left(\bar{X_{i}}-\bar{Y_{i}}\right)}^{2}}\right)$$ (2)

For pathway P, A(P) is the deviation score of the pathway, N is the number of differentially expressed genes in this pathway, X_i_ is the mean expression value of gene i across the subgroups, and Yi is the mean expression value of gene i in the sensitive groups. The normal deviation level of pathway P in a given subgroup was obtained by calculating the decimal logarithm of the cumulative sum of the Euclidean distance of all genes in pathway P. Finally, functional pathways in which specific differences were observed among the various subgroups and the associated regulatory genes were identified by comparing the deviation of each pathway between the different subgroups.

### Resistant biomarker identification

We constructed the protein interaction network using resistance-related genes in common between the functional pathways. The gene degree in network was calculated, which indicates how many genes are directly linked to this gene. Genes with higher values have much more impact on the network as a whole, and could therefore influence the expression of multiple resistance-related genes, so therefore, we treated these genes as important resistance-related biomarkers. The interaction information was integrated from the STRING, HPRD, BioGrid databases. The degree of all nodes was converted using the base 2 logarithm.

### Performance on the validation cohort

The discovery and validation cohorts were treated as the training and test set, respectively. All of the identified resistance biomarkers were treated as features. We trained a decision tree model on the training set and then evaluated the ability of the algorithm to distinguish between patients with different prognosis in the test set using identified biomarkers. To eliminate differences among the platforms, data from the discovery and validation cohorts were treated with discretization. For example, for gene i, the mean value of all samples was μ, and the standard deviation was s. If the expression value of gene i was greater than μ + s, the expression value was set as 1; if the expression value was lower than μ - s, the expression value was set as -1. All other expression values were set as 0. The decision tree model was trained using the discovery cohort and resistance biomarkers and was then used to predict outcomes (relapse and non-relapse) in the validation cohort.

## Results

### Extraction of differentially expressed genes

**Fig 1:**
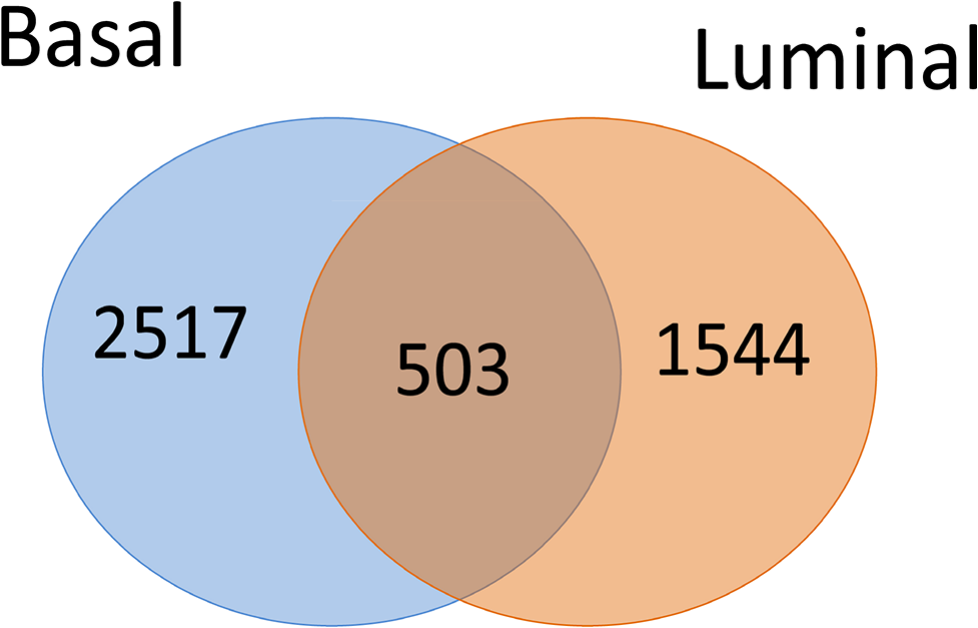
Comparison of differentially expressed genes (DEGs) in Basal and Luminal breast cancer (BC). The left circle (blue) represents DEGs in basal type BC patients, and the right circle (orange) represents DEGs in luminal type BC patients. The overlapping and unique DEGs in two types of BC are shown using a Venn diagram.

We identified a total of 2047 luminal-related differentially expressed genes between the resistant and sensitive patients through random perturbation analysis of the sample data, including 1149 downregulated genes and 898 upregulated genes. A total of 3020 basal-related differentially expressed genes were obtained, including 2087 downregulated genes and 933 upregulated genes. Different subtypes of breast cancer have diverse chemosensitivity and may require different chemotherapy strategies. Therefore, we extracted differentially expressed genes between the resistant and sensitive groups in the luminal and basal subtypes, respectively. The comparison between the two subtypes is shown in Fig 1.

Fig 1 shows a comparison of the genes that were differentially expressed between the basal and luminal-like breast cancer samples. Of these, 503 genes were found in common, accounting for 16% and 24% of the differentially regulated genes in the basal and luminal-like types of breast cancer, respectively. Therefore, the genes in this intersection were stably expressed in these two types of breast cancer and may be involved in the mechanisms of drug resistance shared by multiple breast cancer subtypes. On the other hand, many drug resistance-related genes were specific for only one of the two subtypes, as 84% of the basal-related genes and 76% of the luminal-related genes were differentially expressed in only a single subtype. Therefore, unique drug resistance mechanisms may exist in different subtypes of breast cancer. Identifying the specific mechanisms of drug resistance in these subtypes could provide the basis for personalized therapies in clinical practice.

### Hierarchical clustering

**Fig 2:**
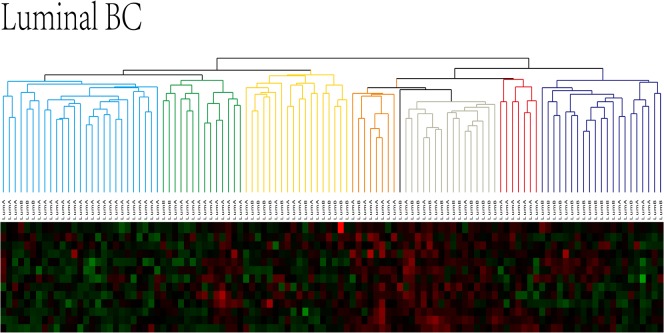
Hierarchical clustering of luminal breast cancer samples. A green-red heat map was used to visualize the clustering results. As illustrated, luminal type BC can be divided into multiple subgroups, indicated with different colors. Both similarities and differences were present between the subgroups. The red and green color key in the heat map represent up- and downregulated genes, respectively.

To identify and validate the existence of different subgroups within a breast cancer subtype, hierarchical clustering was performed using samples of luminal and basal-like breast cancer based on the genes that were identified as differentially expressed in these two subtypes. As there are two subgroups of luminal breast cancer, luminal A and luminal B [17], hierarchical clustering was first performed with the 112 luminal breast cancer samples, based on the 2047 differentially expressed genes, to validate the ability of our method to distinguish specific subgroups within the same subtype of breast cancer (shown in Fig 2).

Fig 2 shows the clustering results for luminal breast cancer. The 112 luminal breast cancer patients were divided into multiple subgroups based on similarities in the expression levels of the differentially expressed genes. There were 27 patients in group 1 (blue), 89% of which had the luminal A type of breast cancer. There were 14 breast cancer patients in group 2 (green), 86% of which had the luminal A type breast cancer. There were 18 breast cancer patients in group 3 (yellow), 61% of which had the luminal B type of breast cancer. There were eight breast cancer patients in group 4 (orange), 88% of which had the luminal A type of breast cancer. There were seven breast cancer patients in group 5 (red), all of which had the luminal A type of breast cancer. There were 21 breast cancer patients in group 6 (purple), 71% of which had the luminal B type of breast cancer. Group 7 (grey) was the mixed type, which consisted of 16 samples, and 90% of the sensitive group samples were in this group. Detailed Luminal patients labels in each subgroups were shown in Table 1.

**Table 1:** Subgroups of luminal breast cancer.

sub-group	sample num	luminal A	luminal B	CR	dominant subtype
**1**	27	24	3	0	luminal A
**2**	14	12	2	0	luminal A
**3**	18	7	11	0	luminal B
**4**	8	7	1	0	luminal A
**5**	7	7	0	1	luminal A
**6**	21	6	15	0	luminal B
**7**	16	5	11	9	luminal B

Table l lists the seven subgroups identified through hierarchical clustering of luminal samples, where “sample num” refers to the number of samples in each subgroup, “CR” is the number of sensitive samples in each subgroup, and “dominant subtype” is the dominant breast cancer subtype in each subgroup.

As shown in the clustering results, nearly all of the samples in the sensitive group were clustered in the same subgroup (group 7), indicating that the expression of these genes exhibited significant gene expression differences between the sensitive group and the drug-resistant group. In addition, the luminal A and luminal B samples were effectively separated and allocated into different subgroups, demonstrating that the proposed algorithm based on differential gene expression was able to effectively identify and distinguish the different luminal subgroups with high accuracy (minimum 61%, maximum 100%).

**Fig 3:**
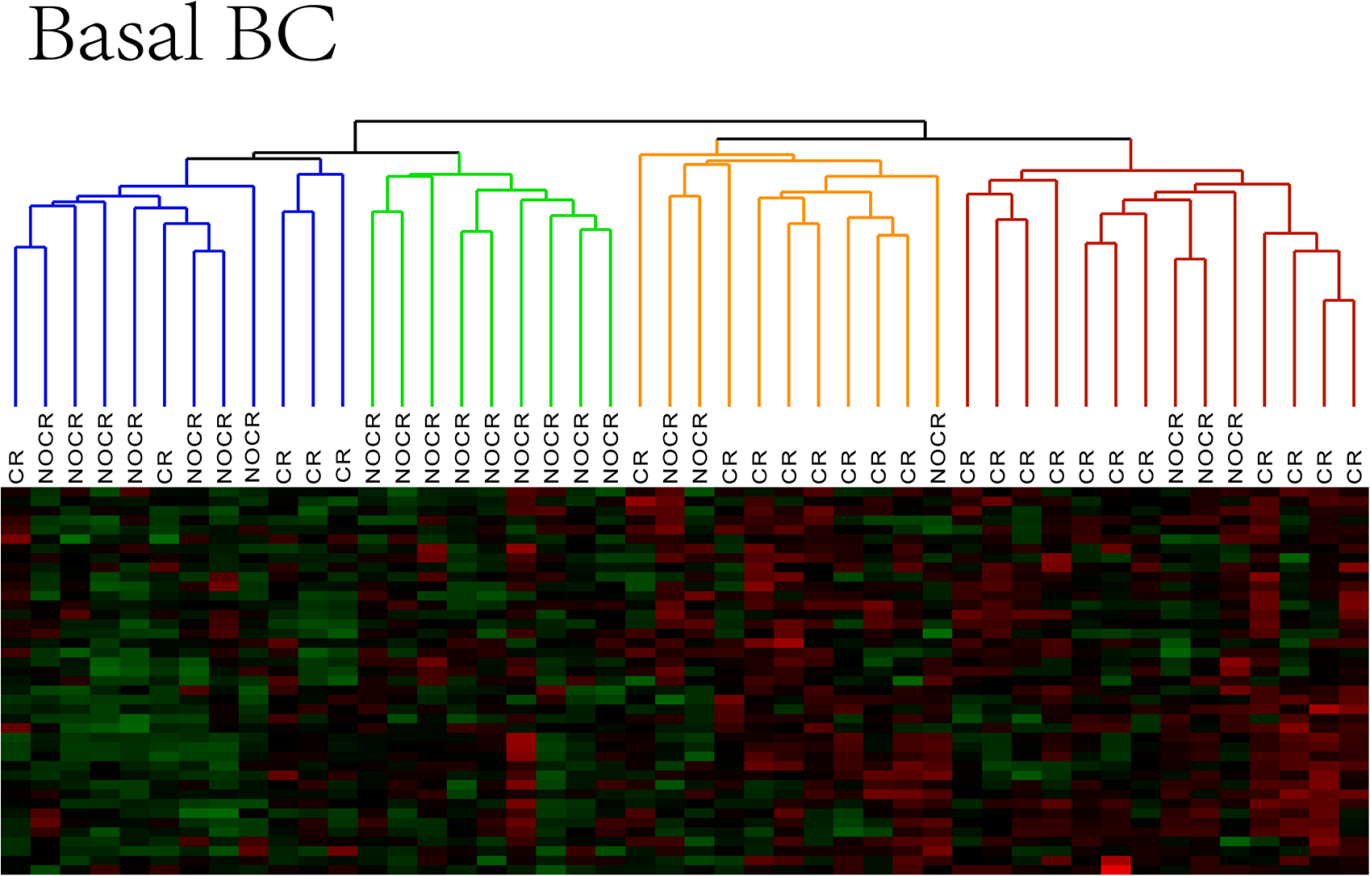
The clustering results for the basal-like subtype. A heat map was used to visualize the clustering results for basal BC. Basal BC can be divided into 4 subgroups, indicated with different colors. CR and NOCR represent sensitive and drug resistant patients, respectively.

As this method could effectively distinguish between the luminal A and B subgroups, this method was also employed for clustering analysis using the basal-like subtype breast cancer samples to identify potential subgroups with different drug resistance mechanisms within this subtype. The clustering results for the basal-like subtype are shown in Fig 3.

Fig 3 shows the clustering results for the basal-like subtype. A total of four subgroups were identified, with group 1 in blue (12 samples), group 2 in green (9 samples), group 3 in orange (11 samples), and group 4 in red (14 samples). The majority of the NOCR and CR patients could be divided into different clusters based on differences at the molecular level. In addition, both NOCR and CR patients could be further subdivided into two distinct subgroups, suggesting that differential drug sensitivity is present even in the same clinical phenotype. Detailed sample information is presented in Table 2.

**Table 2:** Subgroups of Basal BC.

sub-group	sample num	NOCR	CR
**1**	12	7	4
**2**	9	9	0
**3**	11	3	8
**4**	14	3	11

Table 2 shows the distribution of NOCR and CR patients in the four subgroups. CR and NOCR represent sensitive and drug-resistant patients, respectively.

As shown in Table 2, among the four subgroups of basal-like breast cancer, NOCR cases were predominantly found in groups 1 and 2, which were designated as the drug-resistant groups. CR cases were predominantly found in groups 3 and 4, which were designated as the sensitive groups. Therefore, subgroups with distinct molecular profiles were present in both the drug-resistant group and the sensitive group of basal-like breast cancer. Groups 1 and 2 provide evidence for variation in the molecular basis of drug resistance. Indeed, the patients with drug-resistant basal-like breast cancer were divided into at least two different subgroups, indicating the existence of significantly different drug resistance mechanisms. Importantly, this result could explain why resistance to a given drug is only observed in a subset of breast cancer patients with the same cancer subtype, whereas other patients with the same subtype remain sensitive to that drug. Groups 3 and 4 consisted predominantly of drug-sensitive samples. These groups belonged to the same class in the first round of cluster analysis, suggesting that the sensitive group could also be further subdivided into subgroups based on similarities at the molecular level. These subgroup-specific biomarkers could help to create personalized treatments for patients with differential drug sensitivity. To identify these biomarker genes, we allocated genes to subgroups based on expression differences among the subgroups.

### Allocation of specific genes in the subgroups

Four different subgroups were identified among the basal-like breast cancer samples, including two drug-resistant groups and two drug-sensitive groups. The differentially expressed genes for basal-like breast cancer were allocated based on their mean expression values in the four subgroups to yield the specific gene sets for each of these subgroups. The number of genes identified for four groups were 1079, 1203, 1122, 1236, respectively (S1 Table). There were 819 common genes in the intersection of subgroup 1, 2, 3 and 4. The genes in the intersection between these groups exhibited stable differential expression in the drug-resistant group and the sensitive group; thus, they are likely to participate in the regulation of the shared mechanisms underlying drug resistance or sensitivity in these different subgroups. However, genes that did not overlap between these groups likely represent genes that are specific to a given subgroup. These genes only exhibit differential expression in specific subgroups, and therefore, they are likely to be involved in the specific mechanisms of drug resistance unique to each subgroup of the drug-resistant group.

### Functional annotation analysis of subgroup specific genes

**Fig 4:**
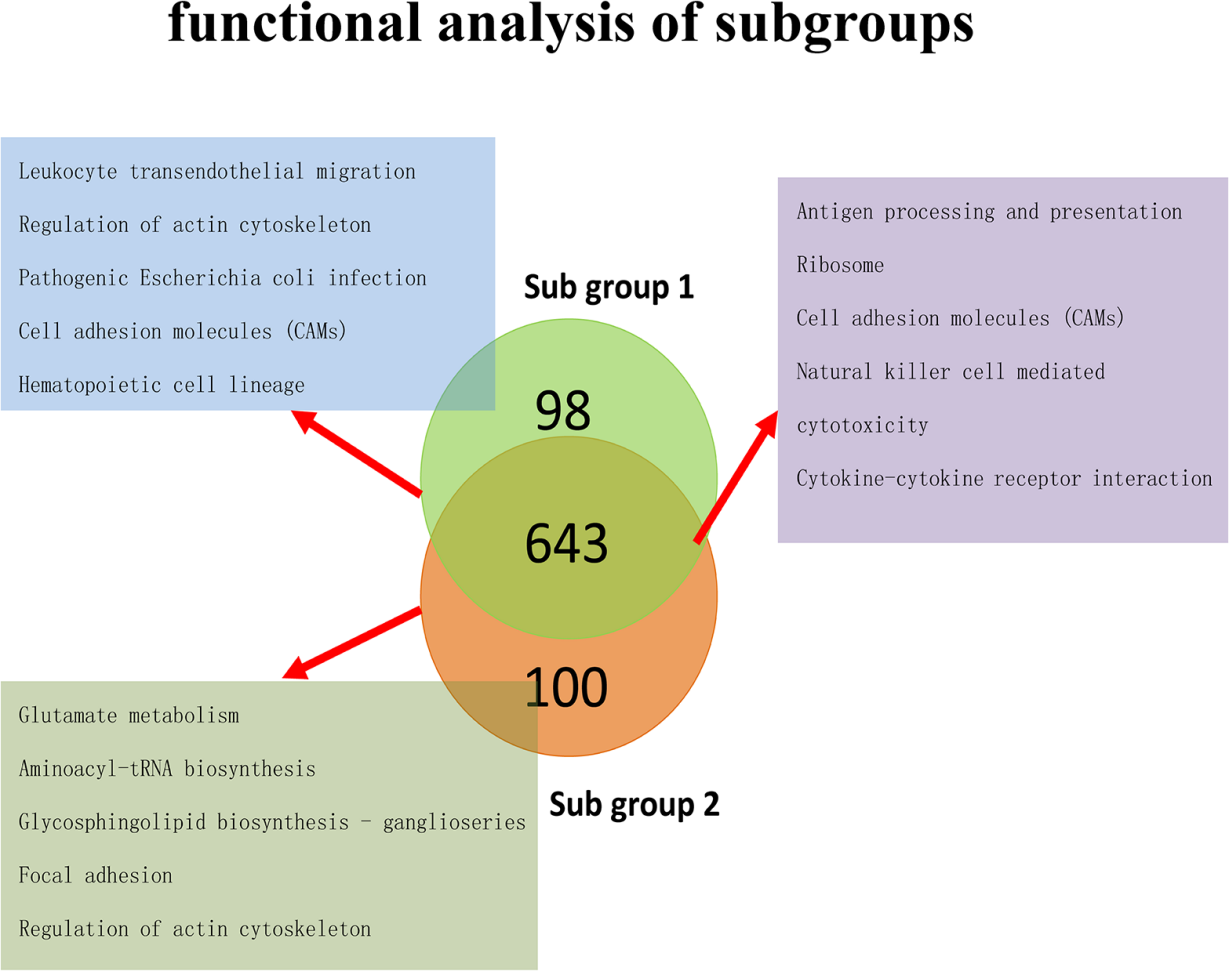
Pathway enrichment analysis. This figure depicts the results of the KEGG functional pathway enrichment analysis with genes specific to subgroups 1 and 2, as well as the genes shared between these subgroups. The pathways in the blue box represent the pathways enriched for the subgroup 1-specific genes, the pathways in the green box represent those enriched for the subgroup 2-specific genes, and those in the purple box represent those enriched for the common genes. Only the top five pathways with the highest significance are listed in the figure; more detailed results are described in S2 Table.

To further study the drug resistance mechanisms that were shared by multiple subgroups or were specific to a single subgroup, KEGG functional pathway annotation analysis was conducted using the genes specific to groups 1 and 2, as well as the genes in the intersection between these groups, as shown in Fig 4 (S2 and S3 Tables).

Comparisons revealed that subgroup 1-specific genes were primarily involved in intercellular signal transduction processes, including the regulation of actin, cell adhesion, hematopoietic cell linkage and leukocyte migration. Subgroup 2-specific genes were primarily involved in the regulation of actin, focal adhesion, and the synthesis and metabolism of amino acids and sphingomyelin. The pathways enriched in the genes that showed overlapping expression patterns in the two subgroups were primarily involved in immune regulatory processes, including antigen presentation, natural killer cell-dependent processes, cytotoxicity effects and cytokine receptor signaling. These findings indicate that the genes that are misexpressed in different subgroups of the drug-resistant basal-like breast cancer group are primarily involved in immune regulation. Therefore, the immune response to chemotherapy agents is likely an important driver of drug resistance. In addition, subgroup 1 and subgroup 2 differentially expressed specific subsets of genes involved in similar pathways, such as actin regulation. These findings indicate that in response to chemotherapy drugs, abnormal connections of the extracellular matrix to intracellular cytoskeletal proteins, resulting from adhesion plaques or actin irregularities, could lead to the blockage of drug absorption by target cells and contribute to drug resistance. Furthermore, genes specific to subgroups 1 and 2 are also critical for processes such as blood cell linkage, leukocyte migration and the metabolism of glutamic acid and sphingomyelin, indicating that abnormalities in blood cell functions or glutamic acid and sphingomyelin metabolism may be important biomarkers for the onset of drug resistance.

Similarly, functional enrichment analysis was also conducted for genes that showed similar expression in subgroups 3 and 4. These genes were stably expressed in both sensitive groups, although their expression levels were quite distinct from those observed in the drug-resistant groups. With respect to the specific drug resistance mechanisms acquired by patients in subgroups 1 and 2, the genes in the sensitive group intersection may be involved in common drug resistance mechanisms that regulate basal-like breast cancer and may in fact represent a common resistance pathway. The enrichment results are shown in Table 3.

**Table 3:** Pathway enrichment of subgroups 3 and 4.

Pathway	Count	p-Value	q-Value
**Jak-STAT signaling pathway**	6	1.41E-05	4.40E-04
**Cytokine-cytokine receptor interaction**	6	2.55E-04	0.003289
**GnRH signaling pathway**	4	4.61E-04	0.004927
**Non-homologous end-joining**	2	9.70E-04	0.008849
**Purine metabolism**	4	0.001703	0.014915
**MAPK signaling pathway**	5	0.002323	0.016957
**Hematopoietic cell lineage**	3	0.003097	0.020551
**Gap junction**	3	0.003737	0.024069
**Nucleotide excision repair**	2	0.009445	0.040375
**Notch signaling pathway**	2	0.010724	0.041937

Table 3 lists significantly enriched pathways. Counts represent the number of DEGs enriched in each pathway. The p values were calculated using the hypergeometric distribution. Q values are the adjusted p values after FDR.

As shown in Table 3, genes stably expressed in the sensitive group were enriched in pathways regulating intercellular interactions, including cytokine receptors, gap junctions, and Notch signaling, as well as multiple other signaling pathways, such as STAT, GnRH and MAPK. Among these pathways, the STAT signaling pathway was the most significantly affected, with six genes being identified, including IL-4R, IL-15RA, STAT2, IL-2RA, CBLB and CSH1. In addition to the STAT pathway, the GnRH [19] and MAPK [20] signaling pathways were also identified as being related to drug resistance in breast cancer.

### Calculation of pathway deviation scores

**Fig 5:**
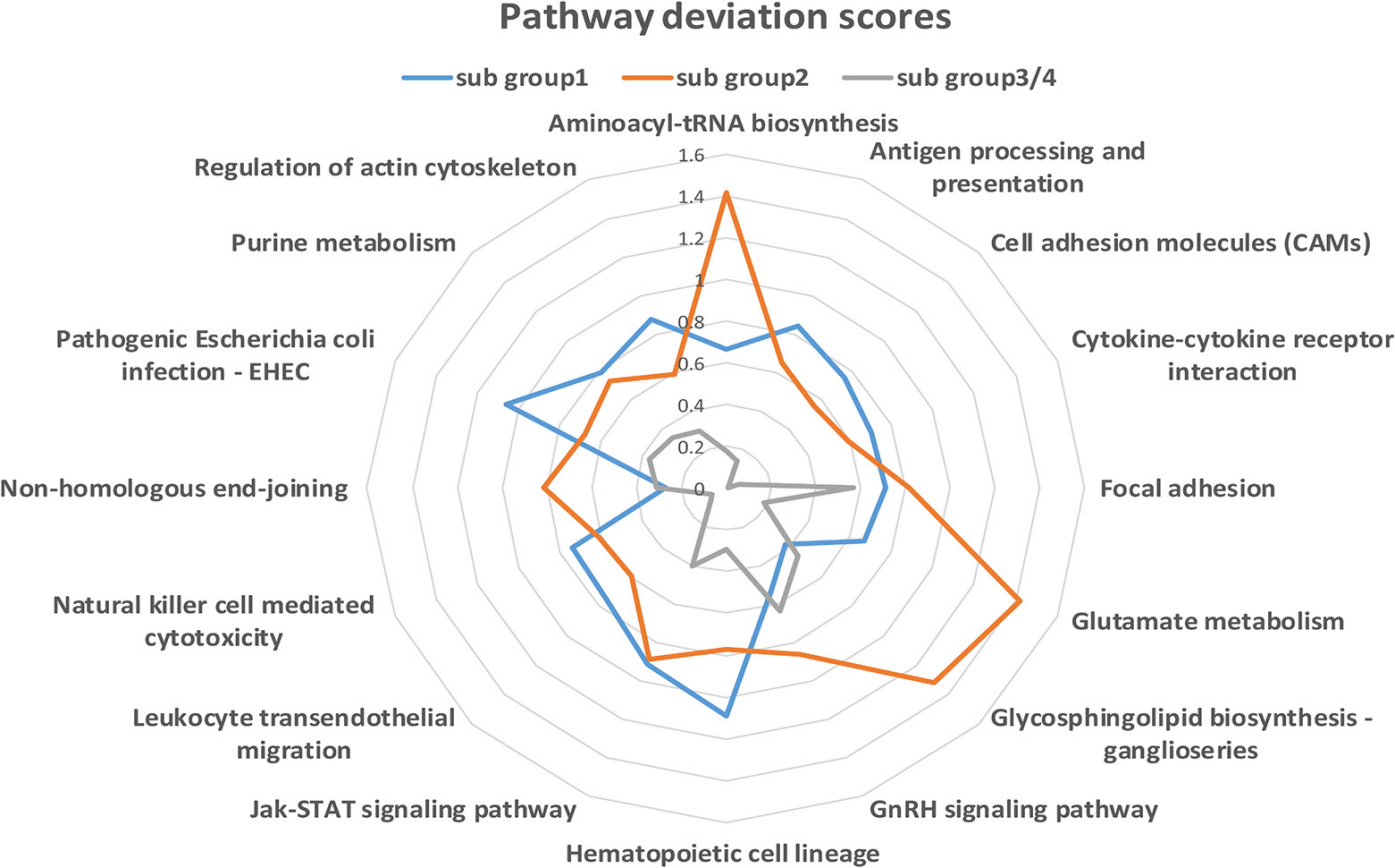
Pathway deviation scores of the subgroups. Subgroups 1 and 2 were marked with blue and red lines. To observe the deviation of subgroup 1 and subgroup 2 from the sensitive range, we also calculated the deviation scores of subgroup 3 and subgroup 4, marked in green (the deviation scores were the same for these two subgroups). The scores of the 16 pathways ranged from 0 to 1.6.

To study functional differences in the specific and common pathways in the various subgroups, the specific and common pathways were integrated for subgroups 1 and 2, and the common pathways were integrated for subgroups 3 and 4. The abnormal deviation scores obtained for each of the 16 pathways in subgroups 1 and 2 and the sensitive subgroups 3 and 4 are listed in Table 4. Pathways showing abnormalities in specific functions were identified by comparing the scores of the two drug-resistant groups (subgroups 1 and 2) and the sensitive groups (subgroups 3 and 4), as shown in Fig 5.

**Table 4:** Deviation scores of the pathways.

Pathway	subgroup 1	subgroup 2	subgroup 3/4
**Aminoacyl-tRNA biosynthesis**	0.67	1.42	0.18
**Antigen processing and presentation**	0.84	0.65	0.14
**Cell adhesion molecules (CAMs)**	0.75	0.56	0.01
**Cytokine-cytokine receptor interaction**	0.7	0.59	0.06
**Focal adhesion**	0.71	0.82	0.57
**Glutamate metabolism**	0.67	1.42	0.18
**Glycosphingolipid biosynthesis—ganglioseries**	0.38	1.32	0.46
**GnRH signaling pathway**	0.53	0.86	0.64
**Hematopoietic cell lineage**	1.09	0.77	0.29
**Jak-STAT signaling pathway**	0.91	0.89	0.4
**Leukocyte transendothelial migration**	0.75	0.59	0.1
**Natural killer cell mediated cytotoxicity**	0.74	0.61	0.07
**Non-homologous end-joining**	0.27	0.81	0.31
**Pathogenic Escherichia coli infection—EHEC**	1.06	0.68	0.37
**Purine metabolism**	0.79	0.73	0.34
**Regulation of actin cytoskeleton**	0.88	0.59	0.3

Table 4 shows the deviation scores of each of the 16 pathways for the different subgroups that were calculated using Formula 1. Higher scores indicate a higher degree of deviation from the normal levels of the pathway, suggesting more obvious abnormalities in pathway function.

As shown in the Fig 5, subgroup 1 exhibited abnormalities in Pathogenic Escherichia coli infection, Natural killer cell mediated cytotoxicity, Hematopoietic cell lineage, Antigen processing and presentation, and Cell adhesion molecules (CAMs), whereas subgroup 2 displayed abnormalities in aminoacyl-tRNA biosynthesis, glutamate metabolism, Glycosphingolipid biosynthesis–ganglioseries and Non-homologous end-joining. Except for aminoacyl-tRNA biosynthesis, all of these pathways that were abnormal in the drug-resistant group have been shown to be involved in the development of drug resistance [21–25]. However, a study by Palaskas N. et al. in 2011 reported that genes related to basal-like breast cancer were highly enriched in the functional pathway of aminoacyl-tRNA biosynthesis [26], indicating a close relationship between basal-like breast cancer and irregularities in this pathway.

### Resistant biomarker identification

**Fig 6:**
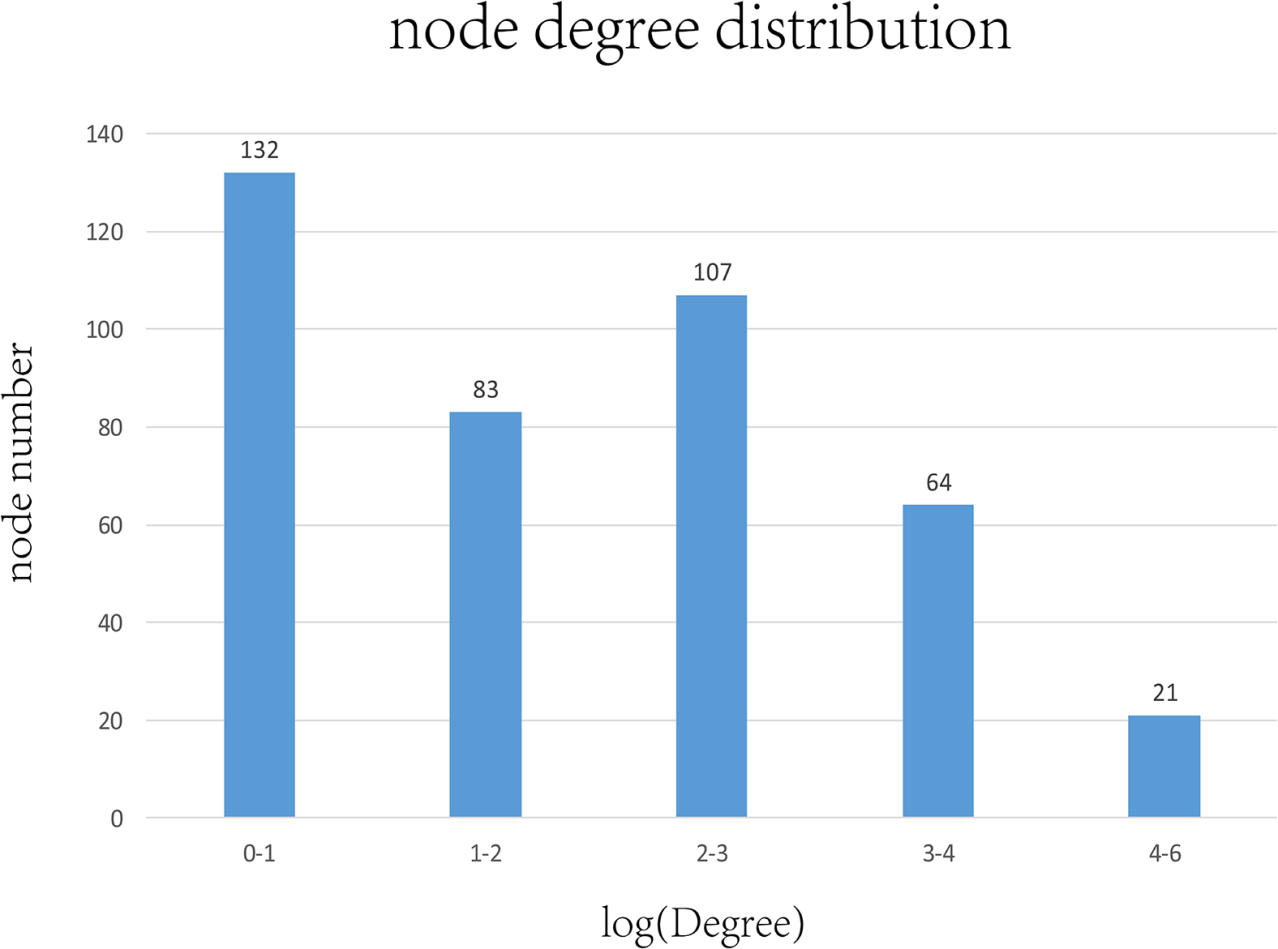
Degree distribution in the PPI network. The network was constructed using candidate genes and common resistance genes. The degree of a gene represents the number of adjacent genes in the network that directly interact with that gene. The degree of all genes was converted using base the base 2 logarithm. Genes with a degree distribution of 4–6 were least common. These genes have a greater impact on the network and are considered to be important drug-resistance markers.

A total of 118 drug resistance candidate genes were extracted from the 16 pathways related to drug resistance. These genes exhibited significant differential expression in at least in one drug-resistant subgroup or one sensitive group. Drug-resistant genes often exert their effects on downstream functional pathways by inducing the abnormal expression of associated genes, eventually leading to decreased sensitivity towards drugs. Therefore, to identify important drug-resistance genes, a PPI network was created for the 118 candidate genes and the 819 common genes based on protein-protein interaction, and the degree distribution was calculated for the nodes. The degree of the nodes was converted using the base 2 logarithm, and the number of nodes that were distributed at different intervals was statistically analyzed. As shown in Fig 6, the number of nodes with a degree distribution at intervals 1–4 in the network was the largest, whereas the number of nodes (21) with a degree greater than 4 was the smallest. Finally, genes that showed interactions with at least 25 drug-resistance candidate genes and common genes were selected as important drug resistance-related biomarkers, which were the gene nodes with a degree greater than 4.7 after logarithm conversion. A total of 9 important marker genes were obtained at the end of the analysis, as shown in Table 5.

**Table 5:** Degree of resistance biomarkers.

resistant biomarker	log(Degree)
**SYK**	6.11
**LCK**	6.11
**PPARG**	5.52
**GAB2**	5.17
**ZAP70**	5.13
**PAWR**	5
**MDFI**	5
**CIITA**	4.75
**ACTA1**	4.7

Table 5 lists the nine resistant biomarkers and their corresponding network degrees. All of these biomarkers have large degree values, indicating they should have a larger impact on drug sensitivity compared with other genes.

Table 5 lists the 9 genes and their corresponding degree distributions after logarithm conversion. These genes are associated with multiple drug-resistance candidate genes or common genes in the network, and therefore play important roles in the development of drug resistance. Verification data were then used to test the effectiveness of these genes in determining the prognosis of TN breast cancer patients.

### Performance of the resistance biomarkers in the validation cohort

The discovery validation cohorts were both treated with discretization, and then we trained the decision tree model using the expression of nine genes from the discovery cohort. The average accuracy reached 83%, representing 95% accuracy for predicting recurrent TN patients and 47% accuracy for non-recurrent TN patients, as shown in Table 6. This result demonstrates the nine identified resistant biomarkers are highly informative for the prediction of relapse due to drug resistance to chemotherapies, although they were less effective for patients who were sensitive to chemotherapies with no relapse. This difference was likely caused by sample imbalance within the validation cohort. As the number of samples without relapse was significantly larger than with relapse (67 vs. 23), the accuracy was lower for the patients without relapse, which had a large number of samples.

**Table 6:** Classification report of the validation cohort.

	precision	recall	f1-score	support
**Recurrence**	0.95	0.64	0.77	23
**Non recurrence**	0.47	0.91	0.62	67
**avg / total**	0.83	0.71	0.73	90

Table 6 A decision tree model was used to predict outcomes in the validation cohort, and the average accuracy reached 83%. The precision, recall, f1-score and support for recurrence and non-recurrence are also included in the classification report.

## Discussion

Breast tissue is composed of highly heterogeneous epidermal cells, including luminal and basal cells, and these two types of cells are considered to be the progenitor cells from which the breast tissue originates [27]. These cell types have significantly different expression markers and biological functions [28], and as a result, different subtypes of breast cancer have remarkably different treatments and prognoses. Clinically, breast cancer is classified based on immunohistochemistry, and most patients diagnosed with basal-like breast cancer (i.e., TNBC) are usually treated with the same therapeutic regimen. However, although some patients are sensitive to this treatment, others develop drug resistance and may suffer relapse. This phenomenon suggests that breast cancers of the same subtype can exhibit markedly different responses to therapeutic agents due to variations at the molecular level. Thus, optimizing treatment outcomes will require personalized therapies. Sensitivity to chemotherapy agents is primarily determined by drug absorption, distribution, metabolism and excretion (ADME), as well as the function of drug efflux pump proteins [29]. By contrast, lower correlations are observed between drug sensitivity and histochemistry types. Therefore, the classification of TNBC patients based on functional protein levels is an urgent clinical need. Personalized therapies based on the sensitivity of the patients to chemotherapy are expected to improve efficacy and reduce unnecessary side effects.

Among TNBC patients, both the drug-resistant and drug-sensitive groups could be further divided into two subgroups, suggesting complex mechanisms underlying drug resistant to clinical chemotherapies. In the two subgroups of the drug-resistant group, the abnormal functions in subgroup 1 were primarily in pathways related to the immune system, such as natural killer cell mediated cytotoxicity, antigen processing and presentation. By contrast, in subgroup 2, the abnormal functions were enriched for pathways associated with the biosynthesis of cell membranes and protein, such as aminoacyl-tRNA biosynthesis and glutamate metabolism. Finally, 9 resistant biomarkers were identified from these aberrant pathways and were validated using the validation cohort, with the mean accuracy reaching 83%.

Central to this study was the use of subgroup-specific genetic markers to determine whether TNBC patients are candidates for routine clinical chemotherapies. If a patient is predicted to be resistant to chemotherapy using this model, other treatment methods should be considered to improve prognosis, such as targeted treatments that avoids toxicity. On the other hand, in the validation cohort, survival “over 3 years” or “less than 3 years” was used to indicate chemotherapy sensitivity or resistance based on concept that non-pCR in TNBC is equivalent to recurrence or poor survival [11]. Therefore, the model established in this study can not only predict the sensitivity of patients to chemotherapies, but it can also determine prognosis, such as risk for relapse.

Two drug-resistant subgroups were identified in this study. These two subgroups exhibited significant differences at the functional level, indicating distinct mechanism of drug resistance between these two types of TNBC patients. Therefore, for patients in subgroup 1, drugs that improve immune functions might be considered to increase drug sensitivity and improve prognosis. For patients in subgroup 2, inhibitors of aminoacyl-tRNA and glutamate synthesis could be used to decrease the proliferative capability of tumor cells. As the 9 resistant biomarkers displayed high-level degree distribution in the PPI network, they broadly regulate multiple drug resistance-related genes and the corresponding downstream pathways. Therefore, these biomarkers should provide new targets for clinical treatments.

This research is highly relevant, as most currently available methods determining breast cancer treatments fail to consider heterogeneity when extracting differentially expressed genes, although a few signatures have been developed to evaluate chemosensitivity of the TNBC patients [30,31]. Therefore, we employed random disturbance to identify specific genes that were only differentially expressed among subgroups to recover individualized chemoresistance genes that would be missed by other methods, in which only the difference between drug-resistant and drug-sensitive groups was examined. Two drug-resistant subgroups were identified with significant differences at the functional level, and the functions of the genes that were misexpressed in each subgroup provide novel insights into the selection of clinical treatment strategies. The nine-gene signature identified in this study can not only predict chemosensitivity, but it can also be used to assess the survival length and the risk of relapse.

This study has several limitations. First, the sample size in the discovery cohort and in the homogeneous validation cohort was limited. In particular, the discovery cohort had unequal numbers of samples of the two prognosis types (67 samples without relapse vs. 23 samples with relapse), leading to a higher predictive accuracy in patients with relapse and a lower predictive accuracy in patients without relapse. Second, the method used to standardize the data from the validation cohort does is not applicable to all published data. For example, the same gene could show large variance between different studies or when different detection methods were used. As a result, to rule out variation in the data across platforms, the validation cohort in this study was selected from the same platform (GPL96), and the data were standardized using the RMA method.

In conclusion, we identified two subgroups of chemoresistant TNBC patients and characterized their personalized abnormal functions. A nine-gene signature was proposed to classify TNBC patients with diverse chemosensitivity and prognoses, and these genes were derived from each resistant subgroup as personalized biomarkers. Therefore, these genes also represent potential therapy targets. By monitoring the expression changes of these genes, it may be possible to optimize therapeutic strategies and dosage adjustments, which could minimize treatment failure and side effects from overdoses. Although further validation and additional research are required, this study points the way towards novel personalized therapeutic strategies.

## Supporting Information

S1 Table Specific genes in subgroups. (XLSX)

S2 Table Subgroup1 pathway enrichment. (XLSX)

S3 Table Subgroup2 pathway enrichment. (XLSX)
